# Assessment of glycemic control using glycated hemoglobin among diabetic patients in Jimma University specialized hospital, Ethiopia

**DOI:** 10.1186/s13104-016-1921-x

**Published:** 2016-02-15

**Authors:** Waqtola Cheneke, Sultan Suleman, Tilahun Yemane, Gemeda Abebe

**Affiliations:** Department of Medical Laboratory Sciences and Pathology, College of Health Sciences, Jimma University, P.O. Box 378, Jimma, Ethiopia; Department of Pharmacy, College of Health Sciences, Jimma University, P.O. Box 378, Jimma, Ethiopia

**Keywords:** Diabetes mellitus, Glycemic control, Diabetic complications, Glycated hemoglobin, Blood glucose

## Abstract

**Background:**

Globally, diabetes mellitus (DM) has risen dramatically over the past two decades and is expected to keep rising for the next 20 years. If uncontrolled it may lead to complications to the patients that could be prevented or delayed. The disease could be diagnosed and monitored by blood glucose and/or glycated hemoglobin (HbA_1_) testing. HbA_1_ can tell long term hyperglycemia of the last 2–3 months period and can predict the risk of diabetic complications; however, the use of glycated hemoglobin test in the country, specifically, in the study area is almost none. Therefore, this study had the aim of assessing glycemic control and describing the risk of complications among diabetic patients using glycated hemoglobin.

**Methods:**

Cross-sectional study was conducted in Jimma University specialized hospital among 148 diabetic patients from May to July 2012. After the study was ethically approved, HbA_1_, random blood sugar (RBS), socio-demographic data and clinical information were collected from every diabetic patients who were willing to participate in the study among patients coming to the hospital for their routine follow up visits.

**Results:**

Even though all the study participants were on diabetes treatment, majority of them were found to be poor glycemic control. It was found out that the mean HbA_1_ and RBS level of the participants were 7.6 % and 280 mg/dL (15.5 mmol/L), respectively. Using HbA_1,5_ 9.5 % of the patients had poor glycemic control and these patients were considered to be at higher risk of developing complications. Among all the study subjects with poor glycemic control, 70.8 % were within 15–30 years of age; 62.3 % were females; 60.8 % were urban dwellers; 67.4 % were illiterate; 69.6 % were with BMI less than 18.5 kg/m^2^, and 61.4 % were taking injectable drugs. Among 136 patients whose clinical history was reviewed, 52.9 % had one or more documented history of major microvascular complications: visual disturbance accounting for 21.3 %, nephropathy 19.1 % and peripheral neuropathy 13.2 %. Eighty-four had poor glycemic control of which 54.7 % had already documented history of one or more complications but the remaining 45.2 % had no documented history.

**Conclusion:**

Even if all of the diabetic patients were on treatment, the mean HbA_1_ level as well as RBS level of the study subjects was above the normal range indicating poor glycemic control. More than half of diabetic patients in the hospital had poor glycemic control and were at higher risk of developing diabetic complications or they already developed the complications. Accordingly we recommended tracing the cause of this poor glycemic control for mitigating the problem.

## Background

Diabetes mellitus (DM) comprises a group of metabolic diseases resulting in hyperglycemia, either because the body does not produce enough insulin, or cells do not respond to the insulin that is produced or both. It can result in acute and chronic complications. Chronic complications harm many parts of the body and majority of diabetes morbidity and mortality are associated to this complication. Hyperglycemia is an important etiologic factor of those complications [1–3].

Recurrent hyperglycemia is the diagnostic feature of DM. More specifically, a patient demonstrating any one of the following is diagnosed as having diabetes: symptoms of diabetes plus random blood sugar ≥200 mg/dL (11.1 mmol/L) or fasting blood sugar ≥126 mg/dL (7 mmol/L) or 2-h plasma glucose during glucose tolerance test (GTT) ≥200 mg/dL (11.1 mmol/L) or glycated hemoglobin (Hb A_1_) ≥6.5 % [4, 5].

For assessing long-term glycemic control in people with diabetes, HbA_1_ is the standard and preferred test. In addition to its use for diagnosis, world health organization (WHO) has endorsed the use of HbA_1_ as a screening test for persons at high risk of diabetes, and more importantly as a test for prediction of the risk of microvascular complications. It is formed by non-enzymatic bonding of adult hemoglobin (HbA) with glucose in the blood, i.e. irreversible glycation of adult hemoglobin. If hyperglycemia stays for long, it leads to glycation of large number of HbA. This glycated HbA circulates for about 120 days which is equal to the lifespan of erythrocyte. Consequently, its value reflects long-term glycemic exposure, representing the average glucose concentration over the last 8–12 weeks [1, 5, 6].

Globally, DM has risen dramatically over the past two decades, from an estimated 30 million cases in 1985–177 million in 2000. It was estimated in 2010 that there were 285 million people with type 2 diabetes making up about 90 % of the cases. Its incidence is increasing rapidly and is estimated that this number will almost be doubled by 2030. The greatest increase in the prevalence is expected to occur in Asia and Africa. In 2010, in sub-Saharan Africa (SSA), above 12 million people had diabetes, and 330,000 people died from diabetes-related conditions. Moreover, access to standard diabetes management in SSA was extremely limited because of insufficient healthcare systems; scarcity of professionals with satisfactory training in diabetes diagnosis and treatment; scarcity or unaffordability of medication, and scarcity of diagnostic tools and other equipment [1, 6–8].

The magnitude of poor glycemic control in diabetic patients in different parts of the world is high. For instance, a study conducted in Malaysia showed 75.3 %, in Spain 45 %, in Jordan 65.1 % and in Ethiopia 94 % [9–12]. Moreover, different researchers had shown that poor glycemic control of diabetic patients leads to microvascular and macrovascular complications. However, lowering HbA_1_ concentrations (by tight glycemic control) significantly reduces the rate of progression of microvascular complications. For instance, dropping HbA_1_ from 9.1–7.3 % reduces the risk of macrovascular disease by 41 %, retinopathy by 63 % and nephropathy by 54 % and neuropathy by 60 %. Every increase in HbA_1_ can increase the cardiovascular event rate by up to 18 % and the microvascular event rate by up to 30 % [13–17].

Researchers around the globe have indicated that there are many contributing factors to poor glycemic control. These include older age, female sex, ethnic variation, drinking alcohol, higher BMI, smoking, longer duration of diabetes, lower physical activity, lack of adherence to diabetes management (such as diabetes self-care management), and many others. A study conducted in Mekelle Ethiopia has also identified that scattered populations, shortage of drugs and insulin and lack of diabetes care team were the major factors behind those serious issues of diabetic control and complications [9, 12, 18].

Another study conducted in Ethiopia showed that there is high prevalence of both acute and chronic complications among diabetic cases, and indicated that only about 1/3 of them receives standard diabetes care. Accordingly the study recommended that effective and efficient prevention and control strategies should be designed and performed in the country. Similarly a study conducted in Jimma indicated that chronic complications were greater than 23 % and overall diabetes management at the hospital were far below any recommended standards. The study proposed that urgent action to improve care for patients with diabetes is mandatory [19, 20].

For provision of standard care for the patients, objective information regarding the magnitude of poor glycemic control is needed; however, studies on the assessment of glycemic control using glycated hemoglobin in Ethiopia are very scarce. To elucidate the few, in 1995 glycated hemoglobin was tested for 102 diabetics who were seen at the outpatient clinic in Gondar in which 78 % of type 1 and 77 % of type 2 diabetic patients were poorly controlled. A study conducted to evaluate the glycemic control and load of complications in 105 diabetic patients at Mekelle hospital in 2007 identified that only 6 % of the patients had good glycemic control. In 2002 another study conducted among diabetic patients at the outpatient diabetic clinic of Jimma University Hospital concluded the presence of overall poor glycemic control [9, 21, 22]. Moreover, another study indicated that none of the diabetic patients in Ethiopia had glycated hemoglobin test. The study also showed that none of the diabetic patients in Addis Ababa had HbA_1c_ determination for their glycemic control and only 21 % of patients had access to blood glucose monitoring at the same health institutions. It was found out that among the patients, only 5 % were able to do self-blood glucose monitoring at home and 51 % of patients didn’t have urine analysis, BUN, creatinine and lipid profile in the previous 1–2 years [23].

Therefore, this study was proposed to fill the gaps observed. Glycated hemoglobin test was used to determine level of glycemic control among diabetic patients and to assess risk of diabetic complications. This study was also aimed to introduce the test method for further routine use in the laboratory of the study area. Moreover, diabetic patients especially those who do not have access to routine monitoring of their blood glucose will benefit from the glycated hemoglobin test which assesses their long term glycemic control status, and this predicts the chance of having diabetic complications.

## Methods

This cross sectional study was conducted among diabetic outpatients in Jimma University Specialized Hospital from 1 May to 31 July 2012. It was aimed to assess the glycemic control status of diabetic patients using glycated hemoglobin test and to determine risk of developing diabetic complication. The hospital is located in Jimma town, Oromia National Regional State at 353 km to the southwestern of Addis Ababa, the capital of Ethiopia. The hospital has 450 beds and is a teaching hospital that gives service to southwestern part of Oromia, part of southern people nations and nationalities, as well as Gambella regions of Ethiopia. The hospital has separate chronic illness referral clinic in which diabetic patients are regularly monitored every Mondays and Tuesdays of the week.

Population for the study were all diabetic patients coming to Jimma University Specialized Hospital diabetic clinic for their regular follow up and willing to participate in the study. Those diabetic patients who were blood transfused within the previous 3 months period were proposed to be excluded from the study.

A total of 148 diabetic patients registered in the diabetic clinic of the hospital and coming consecutively during the study were included. However, the minimum sample size determined using single proportion formula was 87 (using prevalence of HbA_1_ ≥ 7 % = 94 % [9**]**, d-marginal error = 5 %).

About 5 ml of blood was collected for the determination of HbA_1_, RBS, creatinine and urea. HbA_1_ was determined from whole blood but RBS, creatinine and urea were from plasma. Therefore, one EDTA vacutainer tube for the whole blood and one for plasma was utilized from a single venipuncture of a single diabetic patient.

For RBS, creatinine and urea determination one of the two test tubes was centrifuged within 30 min of collection to separate the plasma. Plasma was used to measure glucose as per the ADA and WHO recommendations [6, 16]. GOD-PAP method for glucose measurement using automated Clinical Chemistry analyzer at 450 nm wavelength was utilized. Jaffe reaction method for creatinine and enzymatic urease method for urea determination was performed. Humastar 80 automated Clinical Chemistry analyzer with serial number of 201/79 Ref 16880 in the hospital laboratory was used for the measurement of the blood sugar, creatinine, and urea.

For the HbA_1_ determination, whole blood was mixed with a lysing reagent containing a detergent and borate ions. Elimination of the labile Schiff’s base was thus achieved during the hemolysis. The hemolysate was then mixed for at least 5 min with a weakly binding cation exchange resin. During this time, HbA_0_ (non glycated HbA) binds to the resin. A special resin separator was used to remove the resin from the supernatant fluid which contained the HbA_1_ (glycated HbA). The glycohemoglobin percentage of total hemoglobin was determined by measuring the absorbance of the glycohemoglobin and of the total hemoglobin fraction at 415 nm in comparison with a standard glycohemoglobin preparation carried through the test procedure. CECIL CE 7200 UV-Visble Spectrophotometer with serial number of 137634 and S/W version of R0053 from Wagtech International was used for the measurement of absorbance of the prepared samples and standards. Whole blood specimens were stored for maximum of 2 days at 2–8 °C before analysis. Whole blood specimens are stable for 1 week at 2–8 °C [24, 25].

Other socio-demographic data such as age, sex, weight, height, ethnicity, educational status, duration of diabetes, type of treatment and type of diabetes were gathered from the patients by structured interview and by looking to the clinical data from their charts. The socio-demographic data as well as the laboratory result were documented on the format specifically designed for each of 148 diabetic cases.

Before they were processed, the collected data were cross-checked daily for completeness and then coded, categorized, and summarized. Descriptive statistics and inferential statistical tests using Chi square test and regression analysis were implemented. It was proposed for variables that do not satisfy the assumptions of Chi square test to use the Fisher’s exact test instead of Pearson Chi square test to test the association between the dependent variable and the independent variables. Additionally variables with p value less than 0.25 in bivariate logistic regression analysis were nominated for multivariate logistic regression analysis. p values <0.05 was taken as cut off value for significant association among the dependent and independent variables. Data analysis was performed using SPSS version 16.

The quality of the study was assured by running quality control material every day before running the sample. Moreover, trained data collectors were used to collect the data. Additionally preventive maintenance for the equipment was undertaken and cold-chain was continuously monitored according to the SOP. SOP (for the sample collection, processing and analyzing) were strictly followed so as to assure the quality of the study.

A preliminary study was performed for checking the easiest way of data collection procedure, client satisfaction, and validity of the test kit of glycated hemoglobin before directly going to the real data collection on 5 % of the total study participants.

## Ethical review

The ethical review board of Jimma University reviewed the research proposal and granted the ethical clearance for the researcher to undertake the study. The study participants were informed about the objective, risks, benefits and purpose of the study. Verbal consent was taken from the participants and only those who were willing to participate were included in the study. Confidentiality of information was assured and information was recorded anonymously.

## Limitations of the study

HbA1 test may be altered by factors other than blood glucose (e.g., change in erythrocyte life span, ethnicity) and some conditions interfere with measurement (e.g., selected hemoglobinopathies).

For the random blood sugar testing there are many limitations such as difficulty to have exactly the same postprandial time for every patient under investigation, large biological variability, diurnal variation, sample not stable, numerous factors alter glucose concentrations (e.g., stress, acute illness). Moreover, RBS or FBS is less tightly linked to diabetes complications (than HbA1), and reflects glucose homeostasis at a single point in time.

## Operational definition

1. Diabetic patients having HbA_1_ value greater than 7 % are considered to be at higher risk of developing diabetic complications.
2. Urban = Jimma town and other woreda towns.
3. Poor glycemic control = glycated hemoglobin level ≥ 7 %.
4. Hyperglycemia = blood glucose level above the normal range of 100 mg/dl.

## Result

Though the minimum sample size determined for this study was 87, to increase the validity of the data, a total of 148 diabetic patients, of which 87 (58.8 %) were male were included in the study. The mean (±SD) age of the study participants was 48.5 (±15.7) with the least age of 15 and the maximum 86 years.

From all the study subjects, 65 (43.9 %) were in the age range of 45–60 years; 87 (58.8 %) were males; 92 (62.2 %) were from the urban; 123 (83.1 %) were married; 85 (57.4 %) were Muslims; 95 (64.2 %) were Oromo; 67 (45.3 %) were at primary educational level. All of the study subjects responded that they were on treatment. However, none of the patients was blood transfused within the last 3 months’ time (Table 1).

**Table 1:** Socio-demographic and clinical characteristics of diabetic patients, JUSH diabetic clinic, 1 May 31 July, 2012

Characteristics	Frequency	Percent
Age in years		
15–30	24	16.2
>30 up to 45	34	22.9
>45 up to 60	65	43.9
>60 up to 75	18	12.2
>75 up to 90	7	4.7
Sex		
Male	87	58.8
Female	61	41.2
Residence		
Urban	92	62.2
Rural	56	37.8
Marital status		
Single	16	10.8
Married	123	83.1
Divorced	2	1.4
Widowed	7	4.7
Ethnicity		
Oromo	95	64.2
Amhara	21	14.2
Tigre	2	1.4
Kafa	5	3.4
Others	25	16.9
Religion		
Muslim	85	57.4
Orthodox	51	34.5
Protestant	10	6.8
Catholic	2	1.4
Educational status		
Illiterate	46	31.1
Grade 1–8	67	45.3
Grade 9–12	22	14.9
Diploma and Above	13	8.8
Duration of diabetes		
<1 year	11	7.4
1–5 years	77	52.0
>5 up to 10 years	39	26.4
>10 up to 15 years	15	10.1
>15 up to 20 years	3	2.0
>20 years	3	2.0
Body mass index in kg/m^2^		
<18.5 or underweight	23	15.5
18.5–24.9 or healthy weight	82	55.4
>24.9–30 or overweight	36	24.3
>30 or obese	7	4.7
Current treatment		
Injectables	75	50.7
Oral	68	45.9
Combination of both	5	3.4

Glycated hemoglobin (HbA_1_ %), random blood sugar, creatinine and urea tests were performed for the entire 148 of the study subjects. The mean (±SD) HbA_1_ % of the study subject was 7.6 (±1.9) and the median was 7.5 with the maximum value of 13.5. Among all the study subjects, 88 (59.4 %) patients had poor glycemic control [of which 81(54.7 %) had poor and 7 (4.7 %) very poor] using the result of HbA_1_ (Table 2).

**Table 2:** Glycemic control level by both HbA_1_ and RBS as well as laboratory indicators of renal problem of the study participants, JUSH Diabetic Clinic, 1 May 31 July, 2012

Laboratory test results	Frequency	Percent
Glycated hemoglobin level in percentage		
<7 % (good glycemic control)	60	40.5
7–11 % (poor glycemic control)	81	54.7
>11.1 % (very poor glycemic control)	7	4.7
Random blood sugar (RBS) level in mg/dL		
<70 (hypoglycemic)	1	0.7
70–200 (normoglycemic)l	50	33.8
200–400 (hyperglycemic)	71	48.0
>400 (extreme hyperglycemic)	26	17.6
Creatinine in mg/dL		
Normal (0.5–1.2 mg/dL)	106	71.6
Abnormal (> 1.2 mg/dL)	42	28.4
Urea in mg/dL		
Normal (10–50 mg/dL)	103	69.6
Abnormal (> 50 mg/dL)	45	30.4

Glycemic control status of the study subjects was determined by the result of HbA_1_. According to the result, poor glycemic control was encountered differently among the independent variables of the study subjects. More than half of the study subjects had poor glycemic control when viewed against every variable studied. Majority, [17 (70.8 %)] of those with the age range from 15–30 years, 38 (62.3 %) of female patients, 7 (100 %) of the widowed, 11 (73.3 %) of those with duration of diabetes from 11–15 years, 31 (67.4 %) of illiterate, 16 (69.6 %) of those with BMI less than 18.5 and 46 (61.4 %) of those taking injectable drugs had poor glycemic control. However, poor glycemic control among the categorized independent variables was statistically not significantly different from each other with p < 0.05 (Table 3).

**Table 3:** Glycated hemoglobin (HbA_1_) level versus different socio-demographic and clinical characteristics of study participants, JUSH diabetic clinic, 1 May 31 July, 2012

Socio-demographic characteristics	Glycated Hemoglobin (HbA_1_ in %) level indicating glycemic control level			
	HbA1 < 7 % (good control)	HbA1 ≥ 7 % (poor control)	Total	p value
Age				
15–30	7 (29.1)	17 (70.8)	24	0.636
>30 upto 45	14 (41.2)	20 (58.8)	34	
>45 upto 60	30 (46.2)	35 (53.8)	65	
>60 upto 75	6 (33.3)	12 (66.7)	18	
>75 upto 90	3 (42.9)	4 (57.1)	7	
Sex				
Male	37 (42.5)	50 (57.5)	87	0.556
Female	23 (37.7)	38 (62.3)	61	
Marital status				
Single	7 (43.8)	9 (56.2)	16	0.167
Married	52 (42.4)	71 (57.6)	123	
Divorced	1 (50)	1 (50)	2	
Widowed	0 (0)	7 (100)	7	
Duration of DM in years				
<1	5 (45.5)	6 (54.5)	11	0.613
1–5	29 (37.7)	48 (62.3)	77	
>5 upto 10	19 (48.7)	20 (51.3)	39	
>10 upto 15	4 (26.7)	11 (73.3)	15	
>15 upto 20	1 (33.3)	2 (66.7)	3	
>20	2 (66.7)	1 (33.3)	3	
Educational status				
Illiterate	15 (32.6)	31 (67.4)	46	0.305
Grade 1–8	28 (41.8)	39 (58.2)	67	
Grade 9–12	9 (40.9)	13 (59.1)	22	
≥Diploma	8 (61.5)	5 (38.5)	13	
BMI in kg/m^2^				
<18.5	7 (30.4)	16 (69.6)	23	
18.5–24.9	34 (41.5)	48 (58.5)	82	0.742
>24.9–30	16 (44.4)	20 (55.6)	36	
>30	3 (42.9)	4 (57.1)	7	
Current treatment format				
Injectables	29 (38.7)	46 (61.3)	75	
Oral	29 (42.6)	39 (57.4)	68	0.889
Combination of both	2 (40)	3 (60)	5	

The mean (±SD) random blood sugar (RBS) of this study subjects was 280 mg/dL (±139 mg/dL) or 15.6 mmol/L (±7.7) and the median was 270 mg/dL or 15moml/L. Seventy-one (48 %) of the patients were hyperglycemic and 26 (17.6 %) were extremely hyperglycemic. Fifty (33.8 %) had normal blood glucose level and only 1(0.7 %) had hypoglycemia using RBS testing. Using RBS testing result, poor glycemic control was encountered among 97 (65.5 %) of the patients (Table 2).

According to the result of multivariate analysis, patients with age >30–45 years were 0.26 times less likely in having poor glycemic control [AOR = 0.26, 95 % CI (0.04–1.58)] than patients with age range of 15–30 years. Females were 0.88 times less likely in having poor glycemic control [AOR = 0.88, 95 % CI (0.38–2.03)] than males. Those patients with BMI > 30 were 0.56 times less likely in having poor glycemic control [AOR = 0.56, 95 % CI (0.08–3.85)] than those with BMI < 18.5. Those patients on injectable type of treatment format were 0.51 times less likely in having poor glycemic control [AOR = 0.51, 95 % CI (0.06–4.01)] than those on combination of both oral and injectable format.

However, those who were married were 3.29 times more likely in having poor glycemic control [AOR = 3.29, 95 % CI (0.51–21.11)] than single patients. Those patients having >10 upto 15 years of duration of DM were 5.04 times more likely in having poor glycemic control [AOR = 5.04, 95 % CI (0.69–36.92)] than those <1 year. Those patients with educational level of grade 9–12 were 3.89 times more likely in having poor glycemic control [AOR = 3.89, 95 % CI (0.74–20.42)] than those having diploma and above (Table 4).

**Table 4:** Multivariate analysis of the associations between poor glycemic control using HbA1 and different covariates of study participants, JUSH Diabetic Clinic, 1 May 31 July, 2012

Variables	Patients with poor glycemic control level using HbA_1_ ≥ 7 %		
	No (%)	Crude or (95 % CI)	Adjusted or (95 % CI)
Age			
15–30	17 (19.3)	1	1
>30 upto 45	20 (22.7)	1.82 (0.32–10.34)	0.26 (0.04–1.58)
>45 upto 60	35 (39.7)	1.07 (0.21–5.55)	0.18 (0.03–1.12)
>60 upto 75	12 (13.6)	0.87 (0.18–4.22)	0.39 (0.05–3.12)
>75 upto 90	4 (4.5)	1.50 (0.25–8.97)	0.51(0.03–8.21)
Sex			
Male	50 (56.8)	1	1
Female	38 (43.2)	0.82(0.42–1.59)	0.88 (0.38–2.03)
Marital status			
Single	9 (10.2)	1	1.00
Married	71 (80.7)	1.06 (0.37–3.03)	3.29 (0.51–21.11)
Divorced	1 (1.1)	0.78 (0.04–14.75)	1.53 (0.05–51.58)
Widowed	7 (7.9)	NA	NA
Duration of DM in years			
<1	6 (6.8)	1	1
1–5	48 (54.5)	1.38 (0.38–4.93)	2.01 (0.44–9.24)
>5 upto 10	20 (22.7)	0.88 (0.23–3.36)	1.18 (0.24–5.88)
>10 upto 15	11 (12.5)	2.29 (0.44–11.92)	5.04 (0.69–36.92)
>15 upto 20	2 (2.3)	1.67 (0.115–24.26)	2.89 (0.154–54.22)
>20 years	1 (1.1)	0.42 (0.03–6.06)	0.28 (0.01–12.33)
Educational status			
Illiterate	31 (35.2)	3.31 (0.92–11.85)	3.73 (0.79–17.84)
Grade 1–8	39 (44.3)	2.23 (0.66–7.54)	3.45 (0.78–15.29)
Grade 9–12	13 (14.8)	2.31 (0.57–9.41)	3.89 (0.74–20.42)
≥Diploma	5 (5.7)	1	1
BMI in kg/m^2^			
<18.5	16 (18.2)	1	1
18.5–24.9	48 (54.5)	0.62 (0.23–1.66)	0.72 (0.23–2.27)
>24.9–30	20 (22.7)	0.55 (0.18–1.65)	0.65 (0.17–2.49)
>30	4 (4.5)	0.58 (0.10–3.33)	0.56 (0.08–3.85)
Current treatment format			
Injectables	46 (52.3)	1.057 (0.167–6.716)	0.51 (0.06–4.01)
Oral	39 (44.3)	0.897 (0.141–5.717)	0.60 (0.08–4.84)
Combination of both	3 (3.4)	1	1

Clinical record or chart review was performed for 136 patients regarding the history of complications. Accordingly, 72 (52.9 %) of the patients had one or more history of microvascular chronic complications. Only 6 (8.3 %) patients of them had documented history of complications of less than 3 months duration but the remaining had greater than 3 months. The documented history of chronic complications such as visual disturbance, nephropathy, peripheral neuropathy and others was 28 (20.6 %), 25 (18.4 %), 18 (13.2 %) and 3 (2.2 %), respectively (Table 5).

**Table 5:** Distribution of chronic diabetic complications of the study participants, JUSH Diabetic Clinic, 1 May 31 July, 2012

Type of diabetic complications	Frequency	Percent
Complications history not registered	64	47.1
Visual disturbance	28	20.6
Nephropathy	25	18.4
Neuropathy	16	11.8
Others	3	2.2
Total	136	100

Among those 136 patients whose clinical history were reviewed, 84 had high glycated hemoglobin level out of which 46 (54.8 %) had already documented history of one or more diabetic complications. However, of those whose clinical history were reviewed 52 (38.2 %) had normal glycated hemoglobin level, 26 (50.0 %) of which had already documented history of one or more diabetic complications.

Those [72 (52.9 %)] patients with documented history of diabetic complications were viewed against their socio-demographic characteristics. Accordingly, majority [12 (66.7 %)] of the complications were seen among those with age >60 upto 75 years old, among male sex [44 (55.7 %)] and among the widowed [5(71.4 %)]. Moreover, the complications were mainly observed among those with DM duration of >10 upto 15 years old [10 (71.4 %)], among those with educational status ≥diploma [7 (63.6 %)] and among those who use injectable treatment format [39 (54.9 %)]. However, the presence of this documented history of diabetic complication among the categorized independent variables was statistically not significantly different from each other (p > 0.05) (Table 6).

**Table 6:** Clinical history of diabetic complication status versus socio-demographic and clinical characteristics of the study participants, JUSH diabetic clinic, 1 May 31 July, 2012

Socio-demographic characteristics	Presence or absence of documented history of diabetic complications			
	Present	Absent	Total	p value
Age				
15–30	10 (43.5 %)	13 (56.5 %)	23	0.63
>30 upto 45	15 (51.7 %)	14 (48.3 %)	29	
>45 upto 60	32 (54.2 %)	27 (45.8 %)	59	
>60 upto 75	12 (66.7 %)	6 (33.3 %)	18	
>75 upto 90	3 (42.9 %)	4 (57.1 %)	7	
Sex				
Male	44 (55.7 %)	35 (44.3 %)	79	0.44
Female	28 (49.1 %)	29 (50.9 %)	57	
Marital status				
Single	5 (33.3 %)	10 (66.7 %)	15	0.33
Married	61 (54.5 %)	51 (45.5 %)	112	
Divorced	1 (50.0 %)	1 (50.0 %)	2	
Widowed	5 (71.4 %)	2 (28.6 %)	7	
Duration of DM in years				
< 1	4 (40.0 %)	6 (60.0 %)	10	0.59
1–5	34 (48.6 %)	36 (51.4 %)	70	
>5 upto 10	20 (55.6 %)	16 (44.4 %)	36	
>10 upto 15	10 (71.4 %)	4 (28.6 %)	14	
>15 upto 20	2 (66.7 %)	1 (33.3 %)	3	
>20	2 (66.7 %)	1 (33.3 %)	3	
Educational status				
Illiterate	23 (53.5 %)	20 (46.5 %)	43	0.56
Grade 1–8	30 (47.6 %)	33 (52.4 %)	63	
Grade 9–12	12 (63.2 %)	7 (36.8 %)	19	
≥Diploma	7 (63.6 %)	4 (36.4 %)	11	
BMI in kg/m^2^				
<18.5	9 (42.9 %)	12 (57.1 %)	21	0.78
18.5–24.9	42 (55.3 %)	34 (44.7 %)	76	
>24.9–30	18 (54.5 %)	15 (45.5 %)	33	
>30	3 (50.0 %)	3 (50.0 %)	6	
Current treatment format				
Injectables	39 (54.9 %)	32 (45.1 %)	71	0.31
Oral	32 (53.3 %)	28 (46.7 %)	60	
Combination of both	1 (20.0 %)	4 (80.0 %)	5	

The mean (±SD) blood creatinine and urea tests, the renal function testing parameters, were 1.09 mg/dL (±0.44 mg/dL) and 30.6 mg/dL (±14.5 mg/dL), respectively. High creatinine and urea test result indicating renal problem was encountered among 42 (28.4 %) and 45 (30.4 %) of the patients, respectively (Table 2). Creatinine result was compared with the documented history of complications and 56.1 % of those having history of nephropathy were having high creatinine level. This high creatinine result had statistically significant association with the documented history of nephropathy (Chi^2^ 56.67, df 4, p = 0.00).

## Discussion

It is an established fact that diabetes mellitus is a debilitating disease that can cause complications in those patients whose blood glucose level is not controlled. This study of assessment of glycemic control in diabetic patients has contributed in filling the gap of lack of laboratory test for glycemic control (glycated hemoglobin) and made the test familiar with professionals in the laboratory. Different studies had elucidated that long-term glycemic control is assessed by glycated hemoglobin test. Moreover, long-term glycemic control status of the patients in the study setting was made clear for researchers.

The mean HbA_1_ of the present study is 7.6 % and this is consistent with the 8.5 % report from Jimma [26] but a bit smaller from the 11.3 % report from Mekelle [9]. The mean random blood sugar of the present study subjects is 280 mg/dL which is consistent with FBS of 228.1 mg/dL in Jimma [26], FBS of 228.2 mg/dL in Jimma [27]. It is an already established fact that glycated hemoglobin level greater than 7 %, fasting blood sugar level greater than 126 mg/dL and random blood sugar level greater than 200 mg/dL are considered as poor glycemic control. All the reports indicated above including the report of the present study showed that the glycemic control of the study subjects is poor.

According to different literatures [13–16], those diabetic patients with poor glycemic control, especially with glycated hemoglobin greater than 7 %, are at higher risk of developing diabetic complications. This present study revealed that 59.5 % of the diabetic patients have HbA_1_ test value greater than 7 %. This is consistent with the 52.5 % report from Jimma [28], 63 % report from China [29] but lower than 94.7 % report from Mexico [30]. This poor glycemic control could arise from different reasons such as dietary non-compliance, lack of physical exercise, poor storage and usage of drugs, poor quality of drugs, poor prescription of drugs and any other. These factors could be categorized as patient factors, medication factors, and health care provider (professional) factors.

Different research [9, 12, 18] around the globe indicated that older age, female sex, ethnic variation, drinking alcohol, higher BMI, smoking, longer duration of diabetes, lower physical activity, scattered populations, shortage of drugs and insulin, lack of diabetes care team and lack of adherence to diabetes management are contributing factors to poor glycemic control. In this study; however, it was found out that in every categorized study variables poor glycemic control is more than 50 % except in those having diploma and above in their educational status (poor control is 38.5 %). This poor control in this study is statistically not significantly different among the categories.

Among 136 patients whose clinical history were reviewed, 84 had high glycated hemoglobin level out of which 46 (54.8 %) had already documented history of one or more diabetic complications. However, of those whose clinical history were reviewed, 52 (38.2 %) had normal glycated hemoglobin level, 26 (50.0 %) of which had already documented history of one or more diabetic complications. It is an established fact that HbA_1_ result can predict the risk of complication depending on the previous 3 months status of glycemic control of the patient. Even though researchers identified that increased glycated hemoglobin level could predict the presence of chronic complications, in this study it seems that those with increased HbA_1_ (54.8 %) had similar documented history of complication with those having normal HbA_1_ (50 %). This could be explained by the fact that we found out only 8.3 % of those with documented history of complications had less than 3 months duration of documented complication. This means that about 91.7 % had duration of greater than 3 months period which is above the prediction limit of HbA_1_. Thus the above seeming similarity of documented history is not the real similarity. HbA_1_ can only predict the risk of complication depending on the previous 3 months status of glycemic control of the patient.

Seventy-two (52.9 %) of diabetic patients had one or more documented history of complications, majority of which were visual disturbance accounting 29 (21.3 %), nephropathy 26 (19.1 %) and peripheral neuropathy 18 (13.2 %). This is consistent with report from Jimma [28] that showed 52.5 % of one or more documented chronic complications of which majority of the complications were visual disturbance accounting 33.8 %, neuropathy 29.5 % and nephropathy 15.7 %.

Those documented complications [72 (52.9 %)] were compared against some socio-demographic characteristics of the patients. Even though the categorized independent variables had no statistically significant difference in having complication, the increasing trend with age, with duration of DM, with BMI, is similar with other study conducted in Jimma [28] and in China [29] but in contrary when gender is considered. In this study, though the difference is not statistically significant, male had more recorded complication than female but it was in females in other studies [28, 29]. The reason for this discrepancy in gender needs further exploratory study.

Blood creatinine and urea tests were performed for all the 148 patients. The mean (±SD) of both renal function test parameters was, 1.09 (±0.44) for creatinine and 30.6 (±14.5) for urea. Forty-two (28.4 %) patients had abnormally high creatinine result indicating renal problem. This is in consistent with the already established fact that renal problem result in high creatinine.

## Conclusion and recommendation

In conclusion, even if all of the diabetic patients are on treatment, the mean glycated hemoglobin level (HbA_1_) as well as random blood sugar (RBS) level of the study subjects were above the normal range indicating poor glycemic control.

More than half of all diabetic patients in Jimma University Specialized Hospital had poor glycemic control and were at higher risk of developing diabetic complications and/or even already developed the complications. Therefore, it is very important to look for ways of alleviating this critical situation.

Accordingly it is recommended to trace the cause of such alarming rate of poor glycemic control by conducting different researches so as to alleviate the problem.

## Abbereviations

A_1c_: hemoglobin A1c (the dominating part of minor glycated hemoglobins)
ADA: American Diabetes Association
AGEs: advanced glycosylation end products
BMI: body mass index
DM: diabetic mellitus
EDTA: ethylene diamine tetra acetic acid
FBS: fasting blood sugar
GHb: glycated hemoglobin
HbA_0_: hemoglobin A_0_ (non-glycated hemoglobin)
HbA_1_: hemoglobin A_1_ (glycated hemoglobin)
ICT: information communication technology
JU: Jimma University
JUSH: Jimma University Specialized Hospital
mg/dL: milligram per deciliter
mmol: milimole
nm: nanometer
RBS: random blood sugar
SOP: standard operating procedure
SPSS: Statistical Package for the Social Sciences
